# Team-Based Long-Term Multidisciplinary Inpatient Neurological Rehabilitation After Surgery of Cerebral Meningioma—Outcome and Confounding Factors

**DOI:** 10.3390/brainsci16030263

**Published:** 2026-02-26

**Authors:** Natalie Gdynia, Ingo Haase, Andreas Gratzer, Stefanie Auer, Hans-Jürgen Gdynia

**Affiliations:** 1 Department of Neurology, Fachklinik Enzensberg, 87629 Hopfen am See, Germany; 2 Department of Research, Development and Quality Assurance, Klinikgruppe Enzensberg, 87629 Hopfen am See, Germany

**Keywords:** meningioma, neurorehabilitation, rehabilitation, outcome, tumor surgery

## Abstract

**Highlights:**

**What are the main findings?**

- Following the acute treatment of meningiomas, patients often experience post-operative deficits. Neuropsychological abnormalities are common, while speech difficulties are less frequent.
- Long-term multidisciplinary inpatient neurological rehabilitation appears to be effective in patients following surgical treatment of meningiomas. Statistically significant improvements were found in the areas of grooming, mobility, and climbing stairs. The number of secondary diagnoses has an influence on rehabilitation success. One limitation of our study is that the natural course after surgery without rehabilitation should also be considered.

**What are the implications of the main findings?**

- All patients with postoperative deficits should undergo long-term multidisciplinary inpatient neurological rehabilitation, ideally in a specialized clinic.
- Further studies with a matched control group would be desirable to verify our hypothesis of effectiveness, and optimal intensity, timing, long-term outcome, and modality of rehabilitation should be further investigated.

**Abstract:**

Objective: Cerebral meningiomas are the most common primary intracranial tumors in adults. Treatment of symptomatic tumors is normally surgical; tumors not suitable for surgery can be irradiated. While there is good data on the effectiveness of acute therapy, little is known about the effects of long-term team-based multidisciplinary inpatient neurological rehabilitation. We analyzed the outcome of these patients undergoing neurological rehabilitation. Methods: We performed a retrospective analysis of patients with cerebral meningioma who underwent specialized rehabilitation. We analyzed routine demographic and clinical data; the outcome was measured with the Barthel Index (BI) in patients with a BI of ≤90 on admission. Results: We analyzed 151 patients. Neuropsychological deficits were evident in 93 patients, and 9% had speech disorders. BI increased from 66.8 to 75.2%. Examination of factors influencing treatment success revealed that the number of secondary diagnoses had an influence on the average increase in the BI. No correlation was found for the other independent variables, including age, sex, tumor localization, stage, resection (complete or incomplete), complications, and length of stay. Conclusions: Even given the limitations of our analysis, rehabilitation appears to be effective in these patients. However, further investigations with a matched control group would be desirable to verify our hypothesis. Furthermore, studies regarding optimal intensity, timing, long-term outcome, and modality of rehabilitation are necessary.

## 1. Introduction

Cerebral meningiomas (CMs) are the most common primary intracranial tumors in adults [1]. Although most cases are benign, malignant forms are known. According to a recent study, 80.1% of reported meningiomas in the United States are CNS WHO grade 1, 18.3% are grade 2, and 1.5% are grade 3 [1,2,3]. The incidence of non-malignant meningioma is reported to be the highest among the central nervous system tumors in the United States at 9.73 per 100,000 population. The incidence is increasing due to the aging population [1,2]. Risk factors for the development of CM include heritable genetic polymorphisms in MLLT10 (histone lysine methyltransferase DOT1L cofactor) as well as ionizing radiation, elevated body mass index, methotrexate treatment, and cigarette smoking [1,4,5]. The clinical presentation of CM depends on the tumor location. Typical symptoms are headache due to increased intracranial pressure, focal neurological deficits, and partial or generalized seizures. Cases with personality changes, confusion, and altered level of consciousness can be misdiagnosed as dementia, depression, or other psychiatric conditions [6,7]. The treatment of symptomatic CM is usually surgical, aimed at achieving a complete resection. For cases in which meningiomas cannot be surgically removed, irradiation represents a valid alternative for controlling local growth [8]. Systemic treatment strategies have been associated with limited results and are usually indicated in cases of recurrent or progressive disease not amenable to surgery or radiotherapy [8].

Regarding the natural course of clinically asymptomatic CM, several observational studies have shown a linear growth rate of 2–4 mm/year [6,9], although some tumors show a non-linear, exponential growth pattern or no growth at all [6,9,10]. The natural disease course of symptomatic CM is not well known because these tumors are rarely left untreated [6,11]. The 10-year overall survival for meningioma is 57.1% and 77.7% for patients at a younger age at diagnosis (20–44 years) [6,12]. Higher-grade tumors are more aggressive, with 10-year overall survival rates of 53% for grade II and 0% for grade III patients, despite aggressive therapeutic efforts [6].

While there is good data on the effectiveness of acute therapy, survival rates, and the impact of day of surgery on the outcome [6,8,13,14], little is known about postoperative deficits, the effects of long-term team-based multidisciplinary inpatient neurorehabilitation after discharge from the acute care hospital, and factors that may influence these [15,16]. To close this gap, we analyzed routine demographic and clinical data and the rehabilitation outcome measured with the Barthel Index (BI) of patients with CM undergoing a highly specialized long-term neurological inpatient rehabilitation program.

## 2. Methods

In this retrospective study, we analyzed all patients diagnosed primarily with CM who were treated at our clinic between 2019 and 2024, with the main objective of analyzing the rehabilitation success. The evaluation was conducted in accordance with the requirements of the Declaration of Helsinki. The responsible ethics committee approved the study.

The present study is a subproject of a larger overall project that deals with the outcome after surgical treatment of meningiomas. A paper dealing with the rehabilitation outcome after surgery of spinal meningiomas has already been published [17].

The m&i-Fachklinik Enzensberg is a highly specialized clinic with departments of neurology, orthopedics, and specialized pain medicine. In the neurological department, neurological rehabilitation and early rehabilitation is conducted in accordance with the principles and guidelines of the German Neurological Society, the German Society of Neurorehabilitation, and the German Bundesarbeitsgemeinschaft für Rehabilitation. The multidisciplinary treatment approach includes medical therapy, nursing care, physiotherapy, occupational therapy, speech and swallowing therapy, and psychology. Additionally, the therapeutic team consists of nutritionists, social workers, and other specialists. To identify patients with CM, we used the clinic’s information system.

In all identified patients, routine demographic and clinical data were analyzed (n = 151). To measure the rehabilitation outcome, we analyzed the BI at the beginning and the end of rehabilitation for the subgroup of those with a BI of 90 points or less on admission (n = 53). The reason for limiting the analysis of results to patients with a BI ≤ 90 upon admission is that, naturally, only patients who have measurable deficits upon admission can show measurable improvement.

BI is regularly surveyed in German rehabilitation clinics and is well established and validated [18]. In addition to the total BI score, we also analyzed the BI subitems. Dependency analysis was performed to analyze the influence of gender, tumor location, tumor grade, extent of resection, and surgical complications on the rehabilitation outcome.

Statistical analysis was conducted using IBM SPSS Statistics version 29. The analysis was performed using descriptive statistics (medians, means, standard deviation, percentages), with *t*-tests being used for paired samples, and for dependency analysis, different non-parametric tests were used depending on the requirements of the data (Mann–Whitney U test, Kruskal–Wallis test, Spearman’s rank correlation); a *p*-value < 0.05 was considered significant. Statistical analyses were performed on the entire cohort, as well as on patients with a BI of ≤90 points at admission.

## 3. Results

Between 2019 and 2023, 151 patients with the main diagnosis of CM were treated, of whom 53 had a BI ≤ 90. The demographic and clinical characteristics of the patients are outlined in Table 1.

**Figure 1:**
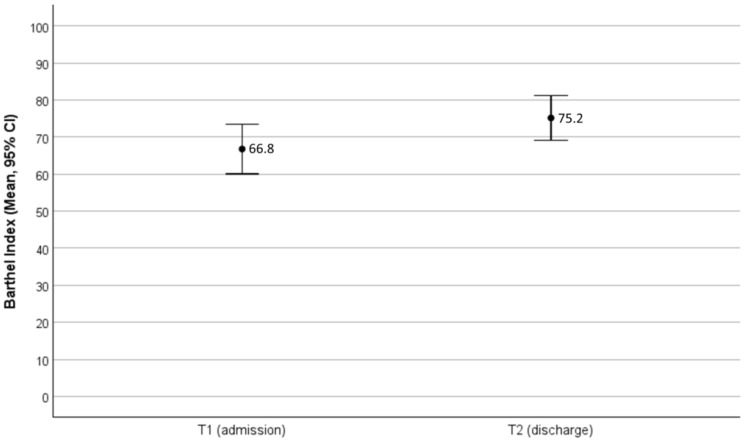
Changes in mean BI during rehabilitation in patients with BI ≤ 90 at admission with 95% confidence intervals. T1 = examination at admission, T2 = examination at discharge, CI = confidence interval.

Figure 1 illustrates the changes in mean BI values during rehabilitation in patients with BI ≤ 90 at admission.

In this subgroup, BI increased from 66.8 to 75.2 points at discharge, which was statistically significant (*p* < 0.001, Cohen’s d = 0.862).

The BI subitem analysis for patients with BI ≤ 90 at admission is outlined in Table 2.

Regarding this group of patients, the majority showed improvements in all segments of the subitems, although only the capability for dressing, climbing stairs, and mobility had a *p*-value that could be considered significant even after adjustments to the significance level due to multiple testing.

In the examination of possible influencing factors on the treatment outcome, such as age, sex, localization (side and region), tumor stage, resection (complete or incomplete), and complications during the operation (see Table 1), a distinction was made between the absolute change in the BI during the inpatient stay and the change per week.

There was no evidence of an influencing factor on the absolute change in BI (all *p*-values ≥ 0.1). If the average change per week of length of stay is considered as a target variable, the number of secondary diagnoses has a moderate influence. All concomitant diseases existing at admission were considered secondary diagnoses.

The fewer the secondary diagnoses at admission, the greater the improvement in BI per week was (Spearman Rho = −0.300, *p* = 0.029).

No influence on the change in BI could be demonstrated for any of the other independent variables tested (see Table 1, *p*-values > 0.1).

## 4. Discussion

Here we describe the postoperative deficits and the effects of long-term team-based multidisciplinary neurological rehabilitation treatment in patients after CM surgery.

So far, little is known about this topic. Greenberg and colleagues investigated the functional outcomes following hospital rehabilitation in patients who survived craniotomy for primary brain tumor excision and compared them with those in poststroke patients [16]. In this retrospective study, 128 patients with CM were analyzed, with a mean age of 59.9 years; there were 62% male patients. On average, patients were admitted to rehabilitation treatment 13 days after meningioma excision. Functional variables during inpatient rehabilitation in CM patients were found to be similar to those in patients with cerebral glioma and stroke. The average Functional Independence Measure (FIM) at admission was 80.07, and at discharge, it was 90.3 in CM patients. Functional gain was 17.9 for meningioma patients, and the average length of stay was 24 days. Of the meningioma patients, 91.7% were discharged to their homes, and 5.4% were discharged to nursing homes. It was found in one study that patients with brain tumors can achieve good functional outcomes with a shorter length of stay compared to that in stroke patients, with the length of stay being 75.4 days [16]. Whereas Greenberg and colleagues used the FIM, we used the BI, including a subgroup analysis to evaluate the outcome. Greenberg and colleagues did not examine the influence of gender, tumor location, tumor grade, extent of resection, or surgical complications on the rehabilitation outcome, yet we did in our study.

A meta-analysis focusing on the rehabilitation outcomes of patients with brain tumors (including meningiomas) found statistically significant improvements in both BI and FIM scores after rehabilitation (standardized mean differences of 0.60 and 0.75, respectively). On average, functional status, as determined by either the FIM or the BI, improved by 36% [19].

In a recent study, Krajewski et al. compared the pre- and postoperative functional status of patients eligible for resection of malignant and non-malignant primary brain tumors to determine the relationship among tumor type, function, and the course of rehabilitation after surgery [20]. In this prospective observational study, 66 patients with non-malignant intracranial tumors were recruited, among whom 21 had CM WHO grade 1 and 3 had atypical CM WHO grade 2. They found that patients requiring rehabilitation after malignant brain tumor surgery generally showed a worse functional status before surgery than did patients with non-malignant brain tumor surgery. The time taken to achieve motor functionality was similar in both groups. These worsened functional outcomes in patients with malignant tumors did not affect the length of stay or the course of rehabilitation. They concluded that patients requiring rehabilitation after malignant brain tumor surgery were more likely to show neurological deficits and have poorer performance and independence before surgery and at discharge compared to patients with non-malignant tumors. Nevertheless, patients with malignant and non-malignant tumors have similar rehabilitation needs. However, in their work, Krajewski et al. did not focus on the distinct influences of specialized long-term multidisciplinary inpatient neurorehabilitation on the patients’ deficits; the duration of rehabilitation was rather short in their analysis, at a mean 11.7 days [20].

In our study, the whole cohort consisted of 151 patients, and the length of rehabilitation was longer, with a mean of 29.5 days. Moreover, 67.5% of our patients were female, which is in contrast to the data of Greenberg [16] and colleagues, who found a predominance of male patients at 62%. In our analysis, 93 out of 151 patients (62%) showed neuropsychological deficits. To us, this seems like a very high proportion. We observed a wide range of deficits in attention, memory, and executive function. A separate paper from our group will describe the exact nature of the neuropsychological deficits and their progression during rehabilitation. Only 9% of our patients had speech or language disorders and required speech therapy. To measure the success of rehabilitation, we analyzed the BI in patients with limitations in activities of daily living, i.e., with a score ≤ 90. In these patients, the BI increased from 66.8 points at admission to 75.2 at discharge, which is statistically (*p* < 0.001) and clinically significant. This improvement, while statistically significant, appears modest and should be interpreted cautiously.

Regarding BI subitems, there were statistically significant improvements in the areas of grooming, mobility, and climbing stairs. In our view, these points are extremely clinically relevant and of great importance as one of the main goals of neurological rehabilitation treatment is to improve patients’ everyday abilities.

In patients with limitations in activities of daily living (initial BI ≤ 90), we found a negative correlation between BI changes per week and the number of comorbidities. This observation is consistent with data in the literature, where comorbidities were found to be possible confounders of length of hospital stay in small studies [20].

In our opinion, it can be assumed that comorbidities have a negative impact on the rehabilitation process due to the resulting increased medical complexity, reduced physical reserve, and competing health priorities of the patients. In surgical patients, it is also known that comorbidities increase the surgical risk, especially in emergency procedures [21].

We were very surprised by the fact that age did not influence BI changes; this should be investigated in further studies. The lack of influence of tumor grade may be due to the fact that the vast majority of tumors included in the analysis were grade 1, and the few grade 2 tumors did not yield statistically relevant results.

Based on our results, long-term specialized inpatient neurorehabilitation appears to be effective for patients with CM, especially regarding mobility, which, in our opinion, is crucial for patients’ quality of life. According to Loomis and Wakasa, we recommend a multidisciplinary team approach including physical, occupational, speech therapy, nursing, and social work to optimize recovery across all affected domains [15]. A multidisciplinary approach is best provided by specialized rehabilitation clinics, so we recommend referring patients to these clinics once acute treatment is complete. Furthermore, strategies should be developed to prevent or treat comorbidities as effectively as possible before and during rehabilitation since these have a significant impact on the outcome.

In addition, a further focus should be on the diagnosis and treatment of neuropsychological deficits, as these are very common and treatable. Zucchella et al. [22] and Locke et al. [23] reported that structured cognitive therapies (e.g., problem-solving) can enhance quality of life and executive function in early postoperative rehabilitation settings. Additionally, long-term studies of skull-base meningioma survivors reveal relatively preserved quality-of-life metrics compared to those of healthy controls, underscoring the potential for sustained neurocognitive recovery [24].

## 5. Study Limitations

Due to the exploratory nature of the evaluation and the numerous comparisons made, the data analysis presented is purely descriptive. Any statistical anomalies should be viewed as potential avenues for further hypothesis generation rather than as confirmation of existing ones.

One clear limitation of our study is that the natural course of the disease after surgery without rehabilitation should also be considered. In our retrospective data analysis, the absence of a control group weakens the strength of the study. However, the natural course of the disease would also be difficult to study in prospective studies, as no patient with postoperative neurological deficits can or should be deprived of neurorehabilitation, and a potential contribution of spontaneous postoperative recovery would be difficult to investigate systematically. Another limitation may be the selection bias inherent in a single-center, retrospective cohort study.

## 6. Conclusions

Despite the limitations of our analysis, it appears that rehabilitation is effective for these patients. These data need to be verified in a larger patient population over a longer period. A prospective study with a matched control group would also help to verify our hypothesis, even if such a study would be difficult to conduct from an ethical standpoint. Furthermore, the optimal intensity, timing, and modality of rehabilitation require further investigation. Moreover, studies addressing long-term functionality, social reintegration, and neurocognitive health in meningioma survivors are necessary.

## Figures and Tables

**Table 1:** Demographic and clinical characteristics of the patients.

Characteristic	All (n = 151)	BI ≤ 90 (n = 53)
Sex		
Male	49 (32.5%)	16 (30%)
Female	102 (67.5%)	37 (70%)
Age (years)	60.2 (23–86)	67.8 (30–86)
Tumor location—side		
Right	59 (39%)	22 (42%)
Left	86 (57%)	27 (51%)
Bilateral	6 (4%)	4 (8%)
Tumor location—region		
Frontal	62 (41%)	23 (43%)
Temporal	28 (19%)	9 (17%)
Parietal	29 (19%)	11 (21%)
Occipital	10 (7%)	2 (4%)
Sphenoid wing	18 (12%)	4 (8%)
Cerebellar	4 (3%)	4 (8%)
Tumor grade (WHO)		
I	128 (85%)	45 (85%)
II	23 (15%)	8 (15%)
Resection		
Complete	133 (88%)	48 (91%)
Incomplete	13 (9%)	4 (8%)
Irradiation *	5 (3%)	1 (2%)
Complication (during/after surgery)		
Yes	21 (14%)	9 (17%)
No	126 (83%)	43 (81%)
Not applicable (irradiation)	4 (3%)	1 (2%)
Number of secondary diagnoses	5.8 (0–23)	7.4 (0–23)
Barthel Index (median)	100 (5–100)	80 (5–90)
Neuropsychological deficits	93 (62%)	28 (53%)
Speech or language disorders	14 (9%)	7 (13%)
Length of stay (days)	29.5 (4–120)	36.3 (10–120)

WHO = World Health Organization, * = including Cyberknife.

**Table 2:** Changes in BI subitems BI ≤ 90 (n = 53).

BI Item	Change		
	Improved	Worsened	*p*-Value
Feeding	9	1	0.021
Transfers	10	2	0.039
Grooming	7	0	0.016
Toilet use	7	2	0.180
Bathing	6	1	0.125
Mobility	18	1	<0.001
Stairs	13	0	<0.001
Dressing	10	0	0.002
Bowels	7	3	0.344
Bladder	5	2	0.453

## Data Availability

The datasets generated during and/or analyzed during the current study are available from the corresponding author on reasonable request. The data are not publicly available due to privacy reasons.

## Abbreviations

BI	Barthel Index
CM	cerebral meningioma
FIM	Functional Independence Measure Score
